# The Efficacy of Phosphate of Lime in Periostitis

**Published:** 1865-01

**Authors:** 


The Efficacy of Phosphate of Lime in Periostitis.—The *Journal de Chimie Medicale* relates two cases of ostetis, attended with intense pain, in which Professor Piorry resorted with much benefit to the exhibition of phosphate of lime. In one patient the tibia and humerus were the seat of a circumscribed tumefaction and severe nocturnal pain. On superficial examination the osseous structures seemed to have preserved their natural consistency, but plessimetric percussion of the affected parts betrayed a loss of elasticity, and an increased sonorousness of the bones. The precedents of the case being of a nature to indicate the presence of the syphilitic taint in the system, the professor prescribed half a grain of protoiodide of mercury night and morning, fifteen grains of iodide of potassium three times a day, and the application of anodyne poultices. This treatment was steadily persevered in for three weeks, but the pain continued with unabated violence, when M. Piorry, taking into account the swollen and softened condition of the bones, conceived that a couple of drachms of phosphate of lime exhibited daily, concomitantly with the iodide of mercury, might possibly prove advantageous. This treatment was therefore instituted, and the issue of the case justified the professor's surmise. In the course of forty-eight hours the osteocopes had much decreased, and disappeared altogether after an interval of a week. The patient being at the same time an anæmic subject, with a small heart, contracted liver, and a weak pulse, which failed when the arm was kept in a raised attitude, tonics and a generous diet were likewise resorted to.

The second patient complained of excrutiating pains in the left temporal region, which were at first attributed to neuralgia of the fifth pair of nerves, and treated accordingly, but without benefit, by the application of blisters dressed with morphia, and belladonna and opium internally. On closer inspection, M. Piorry discovered, on parting the hair, which was very thick and concealed for a time the true nature of the case, a considerable periostic tumor at the base of the parietal bone. In this instance, also, the plessimeter revealed more sonorousness and less elasticity than on the opposite side of the head. The patient, however, contended, and nothing in her previous history disproved her affirmation, that she had never been affected with any symptom of veneral disease. A drachm of phosphate of lime was exhibited night and morning, and the pains decreased in the course of four days, and in a short time a cure was effected.

In both these cases, the reader will observe that the plessimeter was used to discover the softening of the bony structures, and that in the patient whose previous history pointed to syphilis, the use of the calcareous phosphate was in nowise incompatible with the administration of mercurial preparations. We may further add, with the editor of the *Journal de Chimie Médicale*, that the appropriate salt of lime is the combination obtained by the precipitation by ammonia of a solution of the phosphate in muriatic acid; the deposit should be carefully washed, and preserved in a humid condition.—*Dublin Medical Press*, Feb. 24, 1864, from *Jour. de Méd. et Chir.*

